# Book Reviews

**Published:** 1802-10-01

**Authors:** 


### CRITICAL ANALYSIS

OF THE

### RECENT PUBLICATIONS

ON THE DIFFERENT BRANCHES OF

### PHYSIC, SURGERY, & MEDICAL PHILOSOPHY.

---

*Lectures on Comparative Anatomy*, tranflated from the French of G. CUVIER, Member of the National Inftitute, Profeffor in the College of France, and in the Central School of the Pantheon, &c. by WILLIAM ROSS, under the infpection of JAMES MACARTNEY, Lecturer on Comparative Anatomy in St. Bartholomew's Hofpital. 2 vol. 8vo. 1802. London.

THE firft and moft obvious ufe of the fcience of Anatomy is to give man fuch a knowledge of his own ftructure, as to lay a foundation for the fciences of curing internal difeafe and relieving the effects of external accident. Hence the anatomy of the human body has engaged in a peculiar manner the attention of enquirers; and this fubject has been fo thoroughly inveftigated in thofe parts that appear acceffible to mere induftry of refearch, that a new field muft be opened to the ardour of difcovery. This *has* been done by the admirable labours of Haller, Monro, Hunter, Blumenbach, and many other illuftrious contributors to this interefting fcience; and Comparative Anatomy, (which indeed has at no time been entirely neglected) is every day rifing into importance from the boundlefs field which it opens to inveftigation.

Confidered as the ground-work of Phyfiology and Natural Hiftory, Anatomy may truly be regarded as a fublime fcience, and peculiarly fo, that branch of anatomy which compares the organs and ftructure of the human animal with thofe of the animated world that furrounds him.

An extenfive purfuit of comparative anatomy is however within the compafs of but a few; it requires more confiderable opportunities for collecting fubjects than moft perfons can command; it muft be elucidated by numerous fpecimens and vaft mufeums, and it muft fummon contributions from the moft diftant parts of the globe. Such however have been the means of information enjoyed by the author of the valuable work before us; the immenfe Mufeum of Natural Hiftory at Paris, which fupplied fuch copious materials to the labours of the illuftrious Buffon and his worthy coadjutor Daubenton, has liberally opened its treafures to one whofe name ftands among the very higheft of the anatomifts of Europe, and whofe juftly acquired celebrity has attracted a moft numerous and well well-informed clafs of pupils that will not fail to fupport the reputation of the School of Paris.

The work of which the prefent is a faithful tranflation, was drawn up by Cit. Dumeril from the oral demonftrations of the author, Prof. Cuvier, who honors the editor with the appellation of one of his deareft pupils and beft friends, and has given his full and entire fanction to all that it contains, in the following terms: "Having attended my courfe during four years he has collected all my obfervations with fo much accuracy that it would have been difficult for me to have performed the tafk better. I have revifed his manufcript with the greateft care. I have every where fupplied details which could not be conveniently introduced in public lectures. I have rectified fuch ftatements as I had advanced too rafhly. I have added every information connected with thefe lectures that I have obtained fince their delivery, by my diffections and reading. I therefore do not hefitate to acknowledge this work as my own, and to avow all the affertions it contains." We cannot refufe therefore to confider the prefent publication as an accurate abftract of the lectures delivered by the learned author, containing the outline of his plan of inftruction, and the heads of all the valuable information which is delivered by him from the chair.

Prof. Cuvier has prefixed to the work an interefting letter to J. C. Mertaud, Profeffor of the Anatomy of Animals in the Mufeum of Natural Hiftory at Paris, in which he owns his high obligation to this Mufeum and its conductors, and introduces feveral remarks on the ftudy of Comparative Anatomy, and the defign of the prefent work. He mentions it as a circumftance peculiarly fortunate to him that the invaluable opportunities afforded him by his fituation prevented the neceffity of conftantly recurring to written authorities; and certainly, as the prefent work is not a hiftory of the Science, but a fummary of facts, and a view of its actual ftate of improvement, the author's excufe for not diftinguifhing in every place the claims of each difcoverer, will readily be admitted. The character of the feveral writers on thefe fubjects, in Germany, and in our own country, is fketched in the following free and (may we not fay) candid terms. "The greater number of thefe authors are found among a people, who, though celebrated for their inventive genius and indefatigable patience in every kind of refearch, have not always been able to confine within due bounds their defire to difplay erudition, a defire which perhaps proceeds from too much modefty, and a miftaken deference for others.

"Another people, no lefs admirable for their bold views and vigorous profecution of the fciences, feem to have fallen into the oppofite excefs of that which I have juft blamed, by contemning the labours of foreigners, and efteeming, and even confulting, almoft exclufively, the works of their own countrymen. This kind of pride, which is perhaps ufeful in politics, when carried into the fciences, and, above all, in the fciences which depend on facts, tends only to produce contracted ideas, and leads to a barrennefs which forms the character of fome of thefe authors in Natural Hiftory, tory and Comparative Anatomy. I believe I have made uſe of the principal diſcoveries of the modern authors who have treated Anatomy in a phyſiological manner. Stenon, Swammerdam, Collins, Duverney, Petit, Lyonnet, Haller, Monro, Hunter, Geoffroy, Vicq d'Azyr, Camper, Blumenbach, Scarpa, Comparetti, Kielmeyer, Poli, Harwood, Barthez, have furniſhed the data with which I commenced my career; and though I have myſelf reviewed a conſiderable part of theſe data, the glory of diſcovering them is not the leſs due to the celebrated men I have mentioned."

We ſhall now proceed to give a ſummary view of the different chapters of this work, in which, though the real value of each diviſion may be nearly the ſame, there are many that contain only a ſimple anatomical deſcription of parts, which the ſtudent with his ſubjects before him will peruſe with minute attention, and the general reader will be much inclined to turn aſide.

The firſt chapter (or *Lecture,* as the author terms it, though much too copious for a ſingle lecture to a claſs) contains a very clear and excellent ſummary of the general laws of the Animal Economy, divided into the heads of, the Organic Functions; the Structure of the Organs; the Differences of the Organs; the Relations of the Organs; and the Diviſion of Animals founded on the whole of their Organization.

The chapter opens with a view of the ſimple phenomena of life deduced from the decompoſition of organized matter by the laws of common chemical action after the vital principle has ceaſed; but the author acknowledges the inſuperable difficulties that attend a reſearch of this intricate nature, as, however near to the origin of life we carry our inquiries, the ſubject has already enjoyed vital force, and poſſeſſes the germ of the phenomena which life may afterwards develope.

We are ſoon therefore compelled to abandon this hopeleſs path of reſearch, and muſt limit our inquiries into the texture and compoſition of living bodies, and the actual phenomena which diſtinguiſh animate from inanimate ſubſtances; phenomena which the author characterizes by theſe three circumſtances: "origin by *generation*; growth by *nutrition*; and termination by *death*;" a conciſe and accurate definition!

This, however, applies to every thing that has life; but there are other leſs general faculties which indicate organization, but are not the neceſſary conſequences of it, and of theſe the faculty of *sensation,* and that of *voluntary motion,* are the moſt remarkable. We are conſcious that theſe faculties exiſt in ourſelves, and we attribute them by analogy, and from their apparent exiſtence, to a number of other beings, whom, with ourſelves, we include under the general term — *animals.*

The author then proceeds to ſhew in what manner theſe two faculties of ſenſation and voluntary motion are connected with each other, and how they allow of a greater complication of organs than the vegetable kingdom, and this leads directly to the conſideration of the *functions* of living bodies. The

The fecond head or article of this Lecture, contains an interefting general idea of the ftructure of the animal organs, and the proceffes which they are intended to carry on. Thefe are defcribed as they take place in the moft perfect animals, but in proportion as the fcale of beings finks, they fucceffively difappear; and in the loweft claffes of animals we find nothing but what is neceffarily connected with the idea of an animal, namely, a fac, fenfible, moveable, and capable of digefting.

The third and fourth articles contain a review of the differences that fubfift between the correfponding organs of different animals, and their relations with the other organs of the fame animal; and it is here that the more immediate object of the prefent work, *Comparative Anatomy*, commences.

The fifth article completes the fubject of the firft Lecture, or general head, by giving a fummary view of the actual divifion which the author adopts in the claffification of animals. As thefe three articles will give the reader an idea of the plan and manner of the work, we fhall give a fhort abftract of their contents.

Two important diftinctions fubfift in the organs of motion, forming a divifion of all animals; in the firft, the bones form an internal fkeleton, articulated and covered by the mufcles, and in thefe the body is fupported by a ftrong pillar formed of feveral bony pieces placed one above the other, and called the fpine of the back. Animals poffeffing this kind of ftructure are termed *Vertebral Animals*, a clafs which includes all the *Mammalia, Birds, Reptiles, and Fishes.* In the fecond clafs there are no internal bones, but either merely fcales or fhells which cover the fkin, within which are the mufcles, or elfe there is no hard part that can ferve as a lever or point of fupport for the motions of the animal's body. Thefe are the *Invertebral Animals*, including the *Soft Worms, Infects, and Testacea,* and they are either entirely foft, or have their bodies and members envelloped in fcales articulated on one another, or are enclofed in fhells.

The organs of fenfation prefent confiderable variety. Some animals have no apparent nervous fyftem, fuch as the *Zoophytes* and *Polyps*; others, fuch as the *Mollusca*, the *Crustacea, Infects,* and a part of the *Articulated Worms*, have only the brain above the alimentary canal, and have all the remainder of the common bundle of nerves fituated underneath, and contained in the fame cavity with the other vifcera; and laftly, in the *Vertebral Animals*, the common fafciculus of the nerves is fituated entirely in the back, above the alimentary tube, and enclofed in a canal which paffes through the vertebral column.

All the vertebral animals poffefs the fame fenfes as man, and tafte, touch, and perhaps fmell, appear never to be wanting; but the zoophytes and fome other claffes, want the fenfe of fight, and no organs of hearing have been difcovered in fome mollufca and in infects.

The organs of digeftion exhibit likewife very important differences; one of the moft ftriking of which is, that in the greater number number of the zoophytes the inteftines form a fac with only one aperture, which ferves both for the admiffion of aliment and the expulfion of the fæces; whereas, in all other animals, the alimentary fac has diftinct orifices for thefe different offices. The chyle is determined to the body in two different ways; in the zoophytes, and (as the Author thinks) in common infects, the chyle fimply tranfudes through the parietes of the inteftinal canal, and bathes all the interior of the body; but in other animals it is taken up and conveyed by its peculiar abforbent veffels. In thefe latter too, the colour of the contents of the feveral veffels differs; for the vertebral animals have the blood red, and the lymph colourlefs; but the mollufca have all the fluids nearly as tranfparent and colourlefs as lymph.

The very important differences in the circulation of different animals is next noticed, and the diftinction between the *fingle* and *double* circulation; and in connection with this fubject the correfponding differences in the organs of refpiration are confidered, and the apparent total want of them in the zoophytes.

Finally, the varieties in the mode of generation are defcribed; the generation with or without copulation, and the natural hermaphrodite animals; and feveral circumftances relating to growth and fecretion.

The interefting obfervations on the relation of the different organs and functions to each other, and their fitnefs for the circumftances and habitudes of the refpective animals, are next pointed out, but in fo concife and confequent a manner that they are incapable of abridgment.

The divifion, or method of arrangement, follows next, a fubject of confiderable importance; for though in fome of the fingular forms and varieties of animal organization which Nature difplays, fhe feems to fport with the rules of the mere pains-taking claffifyer, the man of genius and enlightened obfervation will often be able to feize on fome of the grand and leading features of the admirable mechanifm of the animated creation, and open the door to the moft interefting and the fublimeft fpeculations.

The arrangement which the Author follows is founded principally upon the leading anatomical features that diftinguifh the feveral parts of the animal world. It appears to be clear, elegant, and as free from perplexing anomalies as the nature of the fubject will admit of. We cannot enter into the minutiæ of this claffification, but fhall only obferve, that the firft grand divifion is that of *vertebral* and *invertebral* animals; the former of which fubdivides, more regularly than the latter, into the *warm-blooded*, containing the mammalia and birds, and the *cold-blooded*, which embraces the genera of reptiles and fifhes. The invertebral animals break into five genera, anatomically confidered, each of which has its diftinguifhing marks laid down with confiderable precifion; they are, the *mollusca, cruftacea, infects,* the *terrestrial worms,* and the *zoophyta*.

Each of thefe genera is again divided into its feveral fpecies, which which we ſhall not here follow: in fact, for the general purpoſes of arranging facts and aſſiſting obſervations, almoſt any claſſification of the animal kingdom, hitherto given, will anſwer the purpoſe with tolerable eaſe; and perhaps the ſtudy of comparative anatomy is not yet ſufficiently advanced to allow of a compleat, luminous, and unexceptionable arrangement. This ſubject is further illuſtrated by very ample tables given at the end of the volume, which will immediately exhibit the diviſion of animals adopted by the author.

The ſecond Lecture is employed in a full general deſcription of the organs of motion; that is to ſay, the bones and muſcles, and is divided into the ſeveral articles of, the intimate nature of muſcular fibre; the ſtructure, compoſition, and growth of bone; the articulation of bone; the mechanical ſtructure of tendons and muſcles; and general remarks on the ſkeleton.

The characteriſtic chemical property of pure muſcular fibre is well known to be its perfect reſemblance with the craſſamentum after the colouring matter has been waſhed away, and this has been termed *fibrine* by the French chemiſts. The author notices the large proportion of azote which it contains, and the ſingular difficulty which occurs in accounting for its formation in herbivorous animals, whoſe food contains no ſenſible proportion of this principle. We do not think the author at all approaches to an explanation of this difficulty by referring it to the action of reſpiration in removing the hydrogen and carbon from the blood, and thereby augmenting the proportion of azote. With regard to the property of irritability, which reſides in muſcular fibre, the author adopts the opinion, now ſo generally received, that this is owing to the minute ramification of nervous filaments, which penetrate muſcle beyond the reach of ſight; and he obſerves, that the animals which have not diſtinct and ſeparate nerves, have no viſible fleſhy fibres, and in them irritability and ſenſibility do not appear to belong excluſively to any particular ſyſtem of organs. Muſcles do not neceſſarily require the preſence of veſſels and cellular ſubſtance, for the muſcles of inſects contain neither, and are only bundles of unadheſive, parallel, and ſimply contiguous fibres; and yet they act with great ſtrength. Colour is not neceſſary to muſcles, as theſe organs are the ſame in the white as in the red-blooded animals. Several remarks on irritability are added; and the following ingenious method, given by Humbolt, for diſtinguiſhing a nervous from a muſcular filament, is mentioned. He employs two needles, one of gold and the other of ſilver, and applies the point of one to the muſcles, and the other to the filament to be examined; on bringing the other extremities of the needles in contact, the muſcle will contract if the filament be a nerve, but not otherwiſe.

The deſcription of the growth of the bones is clear and preciſe. On the progreſs of oſſification, he remarks, that it is unequal both in different animals, and in different parts of the ſame animal. In man, and all the mammalia, the bones of the internal ear are not only only firſt oſſified, but they ſurpaſs all others in denſity and in the proportion of calcareous phoſphat. The bone of the cavity of the tympanum in the cetacea, particularly the whale and the cachalot, is ſuperior in denſity and hardneſs to marble, and appears quite uniform in its ſection, ſhewing no veſtige of fibres, cancelli, or veſſels. In many animals the oſſification is never compleat, as in the large claſs of fiſhes, which from this circumſtance are called the *cartilaginous fiſhes*, or *chondropterygii*.

Several intereſting remarks on horns, ſhells, and the indurated coverings of various animals conclude this article. Shells, he obſerves, are compoſed like bones of calcareous matter, intimately connected with gelatinous matter, but not conſtantly diſpoſed in lamina. In ſome ſpecies, however, there are ſtrata agglutinated to each other like leaves of paſteboard, which certainly increaſe with the growth of the animal, for theſe ſtrata do not all exiſt in the young animal, but only the outermoſt, which is alſo the ſmalleſt. This is the caſe with the muſcle, in which, when young, the ſhell is only a ſingle ſtratum, and therefore is thin and brittle, but is, bulk for bulk, equally firm with the adult ſhell. During growth a ſucceſſion of ſtrata are added on the inner ſurface of the ſhell, each of which extends beyond the edges of the preceding ſtrata, ſo that each operation of this kind adds to the length, breadth, and thickneſs of the ſhell.

Examples of moſt of the kinds of articulation are to be found in human anatomy, and the ſame may be obſerved of the mechanical ſtructure of tendons and muſcles. The tendons of the cruſtacea, however, in the muſcles of the thighs and limbs, differ from thoſe of the red-blooded animals in being hard, elaſtic, and not apparently fibrous. This tendon is often articulated with the ſcaly caſe which it has to move, in the ſame manner as one bone is articulated with another, and is then connected by a membranous ligament. The great claws of the cray-fiſh afford an example of this kind. The tendons of the molluſca are not apparent. This chapter concludes with general remarks on the ſkeleton, and an enumeration of principal points of reſemblance and difference which are found in the various ſubjects of Comparative Anatomy.

Having thus prepared his Readers by a view of the moſt important general features of the intereſting ſubject before him, the author proceeds to the individual deſcription of parts, and the minutiæ of Comparative Anatomy.

This may truly be conſidered as the moſt valuable part of the work; but the very nature of it renders it impoſſible for us to do more than give the contents of the ſeveral chapters, and a ſpecimen of the mode in which this part is executed; for, a regular ſeries of cloſe condenſed deſcription, of all things, the leaſt admits of an abſtract.

In the third Lecture the author deſcribes the organs of motion (that is, the bones and muſcles) of the trunk; in the fourth lecture, thoſe of the anterior extremity or pectoral member; and in the fifth, the poſterior extremity or abdominal member. The plan purſued in theſe chapters is truly *comparative*, as the author firſt deſcribes the parts as they exist in man, and then proceeds to the same or similar parts in the other mammalia, in birds, and in reptiles; and the corresponding anatmoy of fishes is subjoined. Besides the common form of anatomical description, the author takes occasion to introduce several comparative tables illustrative of the subject, such as, of the number of vertebræ in different animals, of the comparative length of the spine in the mammalia, &c. &c.; and he at times increases the interest of the description by observations on the peculiar uses of parts, and their adaptation to the mode of life which the animal is destined to pursue.

In the sixth Lecture, the comparison with the human subject is necessarily dropped, as in it the organs of motion in animals without vertebræ are described This part is, perhaps, more curious than the other, as it is somewhat less familiar to general students, and includes several of those singularities of structure, which, from their novelty, excite surprize and wonder; at the same time it should be remembered, that as it is the most imperfectly known, and the most remote from analogy with our own species, its utility to the common student of anatomy is less than the former part, whatever it may be to him who pursues this difficult path with a view of discovery.

The mollusca are divided into the *Cephalopoda*, in which the head is furnished with tentacula that serve the purpose of feet, as is seen in the sepia or cuttle fish; the *Gasteropoda*, which have the head free and crawl upon the belly, of which the snail is a familiar example; and the *Acephala*, such as the oyster, in which there is no distinct head. It is only the organs of motion in the animals that are here described, those of sense being referred to another part of this work. We shall give part of the author's description of the organs of the cuttle fish, one of the cephalopoda.

"The cephalopoda have eight conical feet, of different lengths, arranged in a circle at the top of the head round the mouth. The animal can turn and bend them in every direction, and fasten itself to bodies by help of the cups or suckers with which they are furnished. The muscles which perform these motions are very numerous.—Below the skin we find a very thin muscle, the fibres of which are united by a loose cellular substance. It accompanies the skin in all its different shapes, and may, perhaps, be regarded as a *musculus subcutaneus*, employed to corrugate the skin, and give greater force to the muscle situated within it, and upon which it acts as a girdle." After describing the other muscles of the foot with its terminating sucker, he adds, "When an animal of this kind approaches any body with its suckers, in order to apply them more intimately, it presents them in a flat or plain state; and when the suckers are thus fixed by the harmony of surfaces, the animal contracts the sphincter, and forms a cavity in the centre which becomes a vacuum. By this contrivance the sucker adheres to the surface with a force proportioned to its area and the weight of the column of air and water of which it constitutes the base. This force multiplied by the number of suckers, gives that by which all or or a part of the feet adhere to any body. This power of adhesion is such, that it is easier to tear off the feet than to separate them from the substance to which the animal chooses to attach itself"

This chapter concludes with the curious organs of motion of the insects, vermes, and zoophytes.

In the seventh and last Lecture of the first volume, the subject of the organs of motion is properly concluded, by a view of the effects resulting from the united action of these organs, whereby the circumstances of standing, walking, seizing and climbing, leaping, swimming, and flying, are produced. Some of the peculiarities in each of these actions possessed by certain classes of animals are also pointed out. Thus, in standing, the structure of birds that enables some of them to remain so long on one leg unfatigued is explained in the following manner: "There are some animals in which certain articulations are maintained in a state of extension, in consequence of their particular form, and the ligaments attached to them. The *stork* affords an example of this. The surface of the femur that articulates with the tibia, has in its middle a depression which receives a projection of the latter bone In bending the leg, this process is lifted out of the depression and removed to its posterior edge. By this motion the ligaments are necessarily more stretched than during the extension of the leg in which the process remains in its socket. These ligaments, therefore, preserve the leg extended in the manner of some springs, without receiving any assistance from the muscles."

The method in which the active muscular exertions of different animals are performed is described with equal clearness, and terminates the volume.

[ To be concluded in our next number. ]

---

*Practical Observations on the Inoculation of Cow Pox, pointing out a Test of a Constitutional Affection in those Cases in which the local Inflammation is slight, and in which no Fever is perceptible: Illustrated by cases and plates.* By JAMES BRYCE, Member of the Royal College of Surgeons, Edinburgh, &c. and one of the Surgeons to the Institution for the Gratuitous Inoculation of Cow-Pox. Edinburgh, 1802, pp 236. 8vo.

THE greater part of this work is a compendium of the principal facts that have been observed on this interesting topic, selected principally from the several publications of Dr. Jenner, to whom the author gives the eulogium which the inventor of Vaccine Inocuation so well merits. After the numerous publications on Cow-pox which have been laid before the world, it would be needless to recapitulate the contents of the present work; it will be sufficient to observe, that the selection is made with elegance and judgment, and that the relative importance of each circumstance is preserved by receiving a proportional share of notice, so that, as a compendium, this publication yields in value to none.

The author, however, does not appear before the public as a mere compiler, but gives some observations which assume a claim to originality. These we shall briefly notice.

Thofe who have attended to the hiftory of this difeafe, will doubtlefs recollect the event of the firft experiments made by Dr. Woodville, in the Small-pox Hofpital, and the number of fevere puftular eruptions that occurred in this place, which feemed at firft to give a very different character to the difeafe from that which Dr. Jenner had reprefented. They will alfo remember, that in fubfequent obfervations, Dr. Woodville made it fully appear from numerous experiments, which it is needlefs to recapitulate, that the matter of fmall-pox does not *hybridize* with that of cow-pox; but that the occurrence of variolous-like eruptions during vaccine inoculation is in all human probality, owing to the effect of a variolous atmofphere operating on the patient, who, in the earlier ftages of vaccine inoculation, is ftill unprotected from the effect of variolous contagion. Thefe obfervations Dr. W. publifhed in 1800, in a feparate pamphlet; and fubfequent remarks by different medical obfervers have purfued this idea a little further, and have fhewn, in a very decided manner, that thefe eruptions are truly variolous, whilft the inoculated puftule remains vaccine. We think, therefore, it was hardly neceffary for the author of the publication before us, after giving the refult of Dr Woodville's firft experiments, to announce his own explanation of them in the following terms. "I am of opinion, that on attentively confidering the circumftances under which it was formed, and comparing thofe with what has occurred to myfelf and others employed in cow-pox inoculation, I fhall be able to evince, that the feverity of the fymptoms was entirely owing to caufes quite unconnected with cow-pox;" when we find the explanation to be fimply, that the eruptive puftules were variolous, whilft the inoculated puftule remained vaccine.

On the fubject of preferving vaccine matter, which has fo often difappointed medical practitioners, the author propofes, as a new method, "To have a fmall phial made for the purpofe, having a long ftopper which reaches nearly to the bottom. This ftopper is ground at the upper part, fo as to fit the mouth of the phial as exactly as poffible, and that part of it which is within the phial is formed into fquare furfaces which are numbered. Upon thefe fquares the virus is lodged, and when dry, is, with the ftopper, put into the phial, where it is very compleatly fecured from the action of the external air."

This method is ingenious, and probably as efficacious as any other. We have already feen it practifed with fuccefs.

The title page of the prefent work promifes *a test of constitutional affection*, a defideratum of the utmoft confequence, and to this part we fhall particularly direct our reader's attention. Cafes will unqueftionably occur, in which, after the whole progrefs of the inoculation is gone through, it ftill remains a doubtful point, whether the fecurity againft future fmall pox contagion is compleat. The author obferves, with great force and juftnefs, that the conducting of inoculation for the cow-pox has been confidered as of fo trifling a nature as fcarcely to deferve the attention of medical men, and hence has arifen much difappointment and want want of fuccefs: for though, *as a disease,* the inoculated cow-pox may be regarded as trifling, yet as a certain preventive of one of the moft loathfome and fatal diftempers that affects the human race, it deferves the moft minute attention. But here it unfortunately happens, that it is much more difficult to afcertain a conftitutional affection in this difeafe than in inoculated fmall-pox, fo that the very mildnefs of the vaccine difeafe, in this point of view, may be confidered as a difadvantage.

In order, therefore, to afcertain this important point, on which refts the whole of the efficacy of the inoculation, the author has applied to the prefent inftance, fome experiments which have been at times made during variolous inoculation. It is well known that if during this procefs, the fame perfon is inoculated every day until the fever induced by the firft inoculation fupervenes, all the other punctures will advance with increafed rapidity in their progrefs; fo that the puncture which has been made only for twenty-four hours, will, at this period, equal in maturity the original one which had been made eight days before.

Purfuing this idea, the author applied it to the vaccine inoculation, and by a feries of accurate, and, in this view, very curious experiments, (for the detail of which we muft refer to the work itfelf) he found that in cow-pox, in which the characteriftic areola takes place about the eighth day, if a fecond inoculation be performed as late as the fifth or fixth day, it will be fo much accelerated in its progrefs as to have an areola formed within a few hours after the firft, increafing with its increafe, and fading as it fades.

This fecond inoculation he alfo found would run a parallel courfe with the firft, whether there was general fever or not, and this is precifely the cafe in which a criterion for determining conftitutional affection is the moft wanted.

The author gives the particulars of fourteen cafes, in which the fecond inoculation was performed; and by varying the time of performing it, a feries of perfectly conclufive and fatisfactory experiments is prefented to the reader, which are alfo illuftrated by two very well executed plates. From thefe it is concluded, "that the moft proper time for performing the fecond inoculation, is about the end of the fifth or beginning of the fixth day from the firft inoculation. If the fecond inoculation be delayed beyond the fixth day, the affection produced by it will be very indiftinct, and of fhort duration; and, if performed at an earlier period than the fifth day, the contraft between the progrefs of the two affections, with regard to duration, will not be fo great as may be thought neceffary. So that, in order to obtain the propofed criterion in the greateft perfection, the fecond inoculation fhould be performed between thirty-fix and forty-eight hours before the areola of the firft inoculation is expected to appear.

"Where the firft inoculation is accelerated or retarded one or two days, as frequently happens, then the fecond inoculation fhould be performed at a more early or late period accordingly."

 The

The author adds, that if the ſecond inoculation is not accelerated, but proceeds in the uſual courſe, it will prove that the firſt was not neceſſary to produce the conſtitutional effect, and therefore that a third ſhould be performed, as a counter proof of the efficacy of the ſecond.

We have thus given the particulars of the author's propoſed plan, as it certainly deſerves the attention of medical men who practice vaccine inoculation. We hardly think, however, that it is fitted for general uſe; partly, becauſe it increaſes the trouble of the operation, inſomuch, as it would require, in many caſes, a ſecond ſupply of vaccine matter to be procured four or five days after the firſt, which in private practice and country ſituations is not always eaſy to be inſured. Likewiſe, if the ſecond inoculation ſhould fail altogether, whilſt the firſt went through its regular progreſs in the moſt ſatisfactory manner, the author would probably aſcribe this failure to a defect in the virus, which unfortunately ſo often diſappoints the expectations of all parties concerned; but would he then ſuggeſt a doubt on the mind of the parents or friends of the patient of the validity of the firſt operation, on account of the failure of the counterproof? or though *he* ſhould not heſitate to pronounce the ſecurity of his patient from future variolous infection, would *they* remain perfectly ſatisfied after they had ſeen the experiment fail, which he had himſelf inſtituted for his and their ſatisfaction?

Laſtly, we may add, that in delicate irritable children, where already one, or perhaps two puſtules are approaching to their ſtate of higheſt inflammation, it might not be altogether ſafe to increaſe the only part of the vaccine diſorder in which any riſk is incurred, namely, the local inflammation.

However, the plan merits every attention, and the author certainly deſerves well of the benevolent cauſe in which he is ſo actively engaged.

---

*An Historical Account of the Discovery and Education of a* SAVAGE MAN, *or of the first Developements, physical and moral, of the young Savage, caught in the Woods near Aveyron, in the Year* 1798; *by* E. M. ITARD, *Physician to the National Institution of Deaf and Dumb, &c. Translated by* Dr. John Reid, 1802, price 3s. 6d.

We have little to learn concerning the manners, the habits, and the faculties of a man in every ſtate of *Society* from the rudeſt to the moſt poliſhed; the indefatigable zeal of travellers has introduced us to the kraals of the Hottentot, and the ſubterranean huts of the Eſquimaux; and the manners of ſavage ſociety have long afforded food for reflection to the civilized philoſopher. But to complete our knowledge of the powers and reſources of the human animal, we muſt take a ſolitary individual of the ſpecies, deſerted by, or deprived of, his natural protectors at an early age, ſeeking the ſame ſhelter as the wild animals of the foreſt, procuring his ſubſiſtence by the unaſſiſted powers of his body, and thus following only his natural inſtincts, forlorn, friendleſs, and ſelf-dependent.

What

What ſpeculative philoſopher could ever venture to propoſe ſuch an experiment? and how great muſt be the chances againſt a child, thus ſituated, ſtruggling through the helpleſs years of infancy!

Such an intereſting ſubject for the moraliſt, the phyſiologiſt, and the man of reflection, has, however, actually occurred; and the inſtance which we have juſt ſuppoſed, is not hypothetical, for all theſe circumſtances appear, on the ſtrongeſt preſumptive evidence, to unite in the perſon of the boy who was brought to Paris about three years ago, under the name of *The Savage of Aveyron.*

The narration which begins this little work, is perfectly ſimple. "A child about eleven or twelve years of age, who had been ſeen ſome time before in the woods of Caune in France, looking after acorns and roots, upon which he ſubſiſted, was met in the ſame place, towards the cloſe of the year 1798, by three ſportſmen, who ſeized upon him at the inſtant he was climbing a tree to evade their purſuit. They conducted him to a neighbouring village, and put him under the care of an aged matron, from whom, however, before the end of a week, he contrived to eſcape, and fled to the mountains, where he wandered about during the ſeverity of a moſt rigorous winter, clad only in a tattered ſhirt. At night he retired into ſolitary places, approaching, as the day advanced, the neighbouring villages; and in this manner he paſſed a vagrant kind of life, till the time in which, of his own accord, he ſought refuge in a dwelling-houſe in the canton of St. Semin." From hence, we are informed, he was removed to two or three different places, during which time he appeared wild, capricious, and impatient of reſtraint, till at laſt he was ſent to Paris, and finally placed under the care of the Author, who is the ſuperintendant of the Hoſpital for the Deaf and Dumb, one of the moſt excellent and benevolent inſtitutions for its extent in that large metropolis.

Here then begins the ſubject of the intereſting hiſtory before us, which is, to relate the manners, habits, bodily conſtitution, and above all, the degree of reaſon ſhewn by this unfortunate foundling, of whom we may ſay, with the Poet,—

> Earth was his bed, the boughs his roof did frame,
> He knew no beverage but the flowing ſtream:
> His food the fruit with which the woodlands teem,
> . . . . . . . . . . . . . . . . . . . . .
> The ſame to him glad Summer or the Winter breme.
> So paſſed his youthful morning, void of care,
> Wild as the colts that through the commons run.

The expectations that were firſt formed on his arrival at Paris, were ſuch as ſtrongly to awaken public curioſity; even perſons of ſuperior underſtanding ſeemed to have expected to find in him a lively, intelligent child, full of activity and obſervation; and they anticipated the pleaſure of obſerving his aſtoniſhment at the ſplendour of the capital.

What was their diſappointment at finding only "a diſguſting, ſlovenly boy, affected with ſpaſmodic, and frequently with convulſive motions, continually balancing himſelf like ſome of the animals in the menageries, biting and ſcratching thoſe who contradicted tradicted him, expreffing no kind of affection for thofe who attended upon him; and, in fhort, indifferent to every body, and paying no regard to any thing."

Hence it was that, though many people of all denominations, at firft flocked to fee fuch a novelty as a *wild boy*, the public curiofity was foon fatisfied; it prefently ceafed to be the *fashion* to vifit the favage of Aveyron, and he might have been abandoned to neglect, if, fortunately for him, he had not found in Mr. Itard, a patient, kind, and truly paternal guardian, who appears to have devoted much of his valuable time to the laborious and almoft hopelefs tafk of foftening the untractable fpirit, and roufing the dormant faculties of this wild and uncouth child of Nature.

The Author proceeds to explain the plan which he laid down, in order to begin the education, civil and moral, of his fingular pupil. The plan is truly philofophical: the fuccefs refulting from the labour of two years has been but partial; enough, however, as it fhould feem, to encourage his kind guardian to perfevere in a courfe, every ftep of which affords fo much matter for interefting reflection.

We fhall not, however, purfue further our account of this little volume, as it will doubtlefs be perufed with eagernefs by all who can feel the intereft arifing from fuch a curious fubject. We cannot help remarking, however, how ftrongly the love of natural objects, and the force of early impreffions recurred at various times to this untaught youth. Who can read without emotion the following paragraph! —" I took him, not long ago, to the Vale of Montmorency. It was a very curious and exceedingly interefting fpectacle to obferve the joy which was painted in his eyes, in all the motions and poftures of his body, at the view of the hills and woods of this charming valley. He fpent two days at a rural manfion; fuch here was the influence of his mind, arifing from the exterior agency of thefe woods and thefe hills, with which he could not fatiate his fight, that he appeared more than ever reftlefs and favage; and in fpite of the moft affiduous attention that was paid to his wifhes, and the moft affectionate regard that was expreffed for him, he feemed to be occupied only with an anxious defire of taking his flight, —rifing from table every minute, he ran to the window with a view of efcaping into the park."

It has been fuppofed that this boy is naturally deficient in his underftanding, and that he can never be made more fagacious than an ideot, who may be trufted to his own guidance in common things. No pains have hitherto fucceeded in giving him the ufe of language, excepting in the pronunciation of one or two fimple founds; however, Mr. Itard alleges with fome force, that he expreffes his fimple wants fo compleatly by figns, as to render fpeech unneceffary. One or two of thefe may entertain the reader. " Is he impatient to dine? He himfelf lays the cloth on the table, and prefents Madame Guerin (his governante) the plates, that fhe may go into the kitchen to fill them. When he dines with me in town, all his wifhes are expreffed expreffed to the lady who does the honour of the table. It is always to her that he addreffes himfelf to be ferved with what he wants. If fhe pretend not to underftand him, he puts the plate by the fide of the difh of which he defires to partake. If this do not produce any effect, he takes a fork and ftrikes it two or three times on the edge of the difh. If fhe ftill neglect him, he lofes all patience, he plunges a fpoon, or even his hand into the difh, and in the twinkling of an eye he empties it all into his plate."

It feems, likewife, that he has not yet learned politenefs, for "a great number of the curious know how, with more natural franknefs than politenefs, he difmiffed them when fatigued with the length of their vifits; he prefents to each of them, and yet without a countenance of contempt, their cane, gloves, and hat, pufhes them gently towards the door, which afterwards he violently fhuts upon them."

The author concludes the work by remarking, that the important period of puberty is juft approaching by the moft unequivocal figns. The phyfiologift will probably expect, that if any material advance is ever to be made in raifing the intellect of this youth to the level of that of his fellow men, it will be effected along with the growth of his bodily powers.

---

*A Practical Synopsis of the Materia Medica,* by the Author of Thefaurus Medicaminum, Vol. I. containing Part I. Materia Alimentaria, and the Firft Clafs of Part II. viz. Evacuantia. 8vo. pp. 323. London, 1797.

We have introduced the mention of this work, though publifhed fo long ago, for the fake of correcting an error at page 564 of our 7th volume. We there mentioned the 2d vol. of this work, as being a Continuation of Thefaurus Medicaminum, which is a diftinct Treatife. The commendation we beftowed on the other parts, may, with propriety, be applied to the prefent.

---

*An Historical Sketch of the Controversy upon Apoplexy, &c.* by R. Langslow, M. D. 8vo. pp. 52, 1802. London.

As the fubject of this work has been very fully laid before the public in our Journal, and even a great part of the work itfelf, (for feveral letters, &c. are here reprinted from the Journal) our Readers will not expect us to give extracts. And as we obferve among our correfpondents, a much greater diverfity of opinion refpecting the pathology of apoplexy than we expected, we avoid giving any other opinion on the prefent work, than that it appears to be as fair a fketch of the Controverfy, as could be expected from a writer who had been fo warmly engaged on one fide.

---

*A Synopsis*

*A Synopsis, and an Explanation of the Synopsis, of Chemical Nomenclature and Arrangement; containing several important Alterations of the Plan originally reported by the French Academicians*; by Samuel Mitchill, M. D Profeſſor of Chemiſtry in Columbia College, &c. pp. 44. New-York, 1801. London.

It is an evil common to all revolutions which overturn eſtabliſhed principles, to go too far. When the French chemiſts thought it right to revolutionize chemiſtry, they created a new language, ſo adapted to the new principles, that the one cannot vary without the other. Now, if chemiſtry had arrived at perfection when the new language was made for it, there would be no other cauſe for regret, than its rendering all previous works obſolete. But when every day preſents us with ſome new diſcovery, or the correction of ſome error that affects the elementary bodies, or the firſt principles, it is to be lamented that an entire new language ſhould have been propoſed ſo early. We think the temperate *reformation* adopted by the admirable Kirwan, would have been preferable to a *revolution*. The revolution, however, has been received in Europe, and the new Nomenclature is become familiar to us; but Dr. Mitchill is not entirely ſatisfied with it; and in the work before us, propoſes a few new terms. We think that the progreſs of Science is retarded by frequently changing the language of it; and we ſhall think it a cauſe of regret, if America and England ſhould uſe different languages in the ſcience of chemiſtry. As we can give no intelligible account of this work, without giving the Synopſis itſelf, we ſhall content ourſelves with mentioning a few of the names propoſed to be uſed inſtead of the current ones. For Caloric, Anticrouon; Hydrogen, Phlogiſton; Azote, Septon. The other terms differ little from thoſe in general uſe.

---

*The Medical Quærist and Investigator*, by W. P. Russel. 8vo. pp. 13. London, 1802.

The deſign of this ſhort pamphlet is to inſtruct the young ſtudent in medicine how to make the inquiries neceſſary to inform him of the true nature and danger of any diſeaſe. It will alſo much aſſiſt any patient in the country to draw up his own caſe for the purpoſe of conſulting a practitioner at a diſtance. As the price is ſmall, we ſhall be ſurpriſed if every medical ſtudent does not poſſeſs himſelf of it.

MEDICAL

